# Experimental investigation on True Triaxial Deformation and Progressive Damage Behaviour of Sandstone

**DOI:** 10.1038/s41598-019-39816-9

**Published:** 2019-03-04

**Authors:** Zhaolin Li, Lianguo Wang, Yinlong Lu, Wenshuai Li, Kai Wang, Hao Fan

**Affiliations:** State Key Laboratory for Geomechanics and Deep Underground Engineering, China University of Mining and Technology, Xuzhou, Jiangsu 221116 People’s Republic of China

## Abstract

Studying the true triaxial deformation characteristics and progressive damage behavior of sandstone is of great significance for the stability control of roadways. Both the conventional triaxial test (CTT) and the true triaxial compression test (TTT) were conducted for sandstone to investigate its deformation characteristics and the variation laws of volume strain during the progressive damage process under different confining pressures. The conducted experiments showed that both the axial and lateral strains of the rock prior to failure under CTT conditions increased with increasing confining pressure. However, with increasing intermediate principal stress (*σ*_2_) under TTT conditions, both the axial strain, and the lateral strain (*ε*_2_) gradually decreased, and the lateral strain (*ε*_3_, expansion) first slow down and then accelerated. Moreover, the anisotropic characteristics first gradually weakened and then enhanced. The variation of the volume strain increment and the volume strain rate of rock combined with the acoustic emission activity and a three-dimensional rock theoretical model with microcrack defects were analyzed in detail. During the stable crack growth stage III, the volume strain increment and volume strain rate increased with increasing confining pressure under CTT conditions, while they decrease after the initial increase with increasing *σ*_2_ under TTT conditions. During the unstable crack growth stage IV, the volume strain increment increased sharply, while the volume strain rate gradually slowed down with increasing confining pressure under CTT conditions. The internal cracks of the rock were gradually suppressed and the lateral expansion was gradually constrained. The volume strain increment first increased followed by a decrease, and the volume strain rate gradually slowed down after a noticeable acceleration with increasing *σ*_2_ under TTT conditions. The internal micro-cracks gradually evolved from inhibition (in the planes parallel to plane 1–2 and plane 2–3) to accelerated expansion (the planes along the *σ*_2_ direction), and the lateral deformation first weakened and then strengthened.

## Introduction

Rock engineering for applications such as roadways is one of the basic projects of national economic construction and a vital support for the development and progress of society. However, during the excavation process, disasters such as deformation and failure severely affect the stability of the surrounding roadway rock^1^. The roadway research gradually realized that the stress state of rock exerts a very important influence on the mechanical behavior of the surrounding rock. In the actual roadway engineering, the rock mass is closely related to the three-dimensional (3D) stress state. By studying the deformation laws of rock under the 3D-stress state, the deformation and failure mechanism of the roadway can be obtained, which is of great significance for the prevention of disasters and the guidance of project implementation.

Currently, a large number of studies by researchers on deformation characteristics of rocks under 3D stress conditions are available^2,3^. Yang *et al*.^4^ analyzed the effects of different confining pressures on the rock deformation behavior using conventional triaxial tests. Peng *et al*.^5^ studied the post peak strain parameters by conducting triaxial compression tests on coarse marble. Liang *et al*.^6^ investigated the mechanical properties of coarse-grained light-colored granite under conventional triaxial compression, and furthermore examined the effect of rock size on strain and the energy characteristics at different stages. However, these studies on rock deformation behavior mainly focused on the conventional triaxial stress state (*σ*_1_ > *σ*_2_ = *σ*_3_), the influence of the intermediate principal stress was ignored. Since the axial stress was only applied to cylindrical rock, the rock was in an axisymmetric stress state, and the general stress states were not represented (*σ*_1_ ≠ *σ*_2_ ≠ *σ*_3_).

A large number of *in-situ* stress measurements showed that the 3D stress state of the rock mass tends to be significantly anisotropic (*σ*_1_ > *σ*_2_ > *σ*_3_) in engineering^7,8^. True triaxial tests can apply the three different principal stresses independently on each side of rock, which can more accurately simulate natural stress states. During recent decades, with the development of true triaxial machines, a large number of studies focused on true triaxial compression experiments^9^: Mogi^10,11^ successfully developed the 1^st^ true triaxial machine in the world, studied the mechanical behavior of dolomite under true triaxial compression tests, proved the existence of the intermediate principal stress effect of rock, and introduced the criterion of octahedron strength. Michelis^12^ pointed out that the intermediate principal stress effect is an important property of rock materials. However, current research on true triaxial compression mainly focuses on the effect exerted by intermediate principal stress on both rock strength and failure^13,14^, and the strength criterion has also been established^15,16^. Furthermore, most research objects are granite, marble, or other hard rocks. Because of their hard texture, scholars tend to be more concerned about the strength and the failure criteria of rock, while comparatively soft rocks, such as sandstone, remain largely unstudied.

Underground projects such as roadways are mostly undertaken in sedimentary structures that consist of sandstone-filled channels, where deformation problems in the roadways are more prominent. By investigating the true triaxial deformation characteristics of sandstone, the working mechanism and the deformation law of sandstone during the actual stress state can be understood effectively. This provides important engineering significance for the treatment of geological hazards of roadway surrounding rock. To date, the true triaxial deformation characteristics have not been extensively investigated. Haimson *et al*.^17,18^ conducted true triaxial compression tests and reported that the elastic stress-strain range is extended by the intermediate principal stress; thus, the initial segment of destruction was delayed. A true triaxial compression test was conducted by Feng *et al*.^19^ and only the stress-strain relationship of the rock was provided. The axial stress-strain relationship of rock has received more attention while both the lateral and volume strain of rock are often overlooked. The rock volume strain reflects the yield and failure characteristics of rock samples from different perspectives. Moreover, in the actual roadway excavation, the lateral expansion of rock mass plays an important role with regard to geological disasters such as deformation and collapse of roadways. Through research on the regularity of lateral and volume strain of rock mass, references can be provided for controlling the deformation of roadway.

Therefore, a series of conventional triaxial and true triaxial compression tests for sandstone was conducted in this study using a self-developed true triaxial compression test system, combined with an acoustic emission (AE) testing technique^20,21^. The deformation characteristics in the three principal stress directions and the variation laws of volume strain under different confining pressures during the progressive damage of rock were analyzed to uncover the root cause of variations in true triaxial deformation.

## Experimental Methodology

### True triaxial electro-hydraulic servo test system

**Figure 1:**
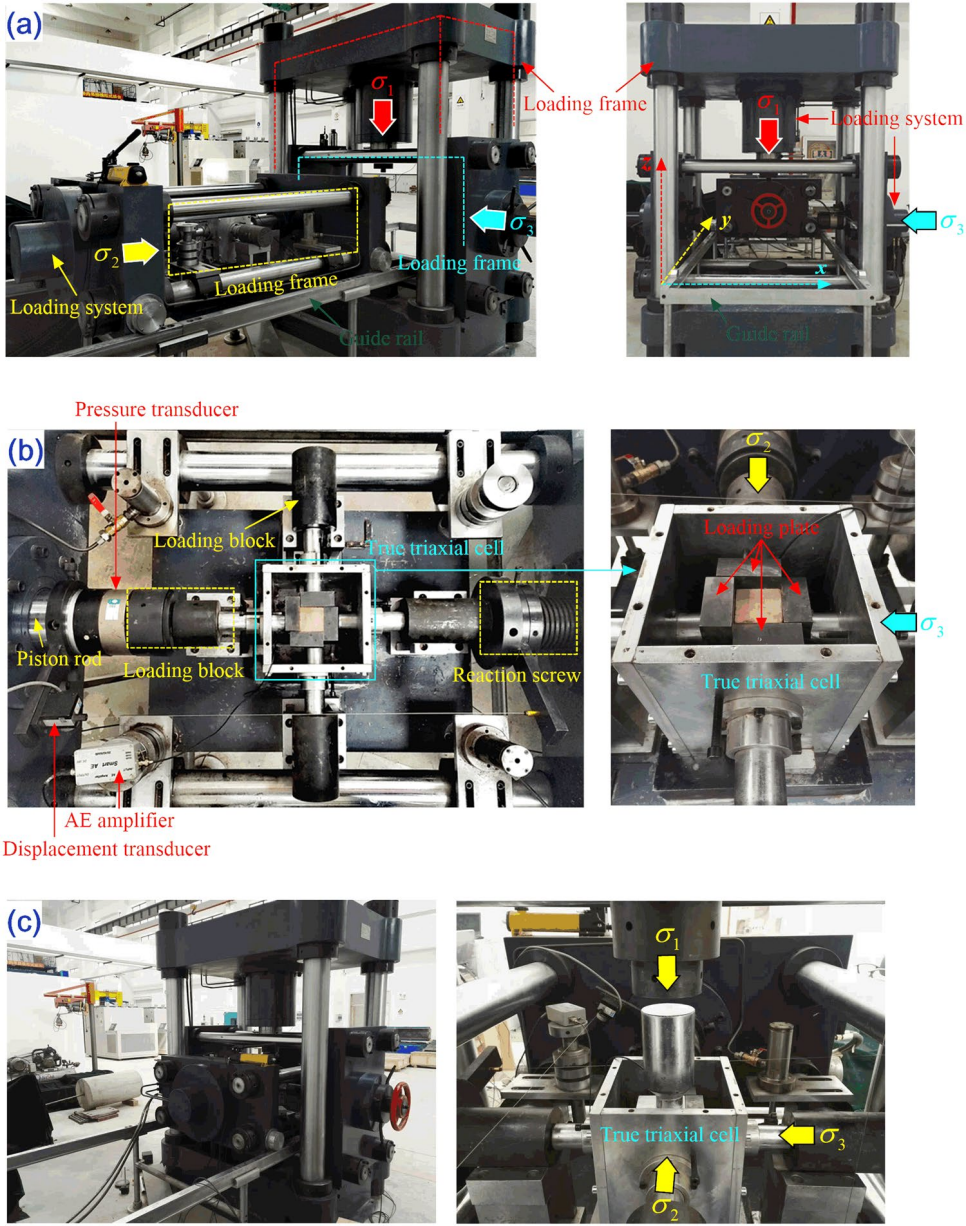
True triaxial compression test system: (**a**) Testing machine frame; (**b**) internal structure of the true triaxial compression test system; (**c**) adjusted true triaxial test system and the three-dimensional position of true triaxial cell.

The tests presented in this study were conducted using a self-developed true triaxial compression test system (see Fig. 1). This test system consists of a three-dimensional servo control loading system (*σ*_1_, *σ*_2_, and *σ*_3_), a true triaxial cell, an automatic acquisition system, and an AE monitoring system. Each of the three-dimensional servo control loading systems adopts the rigid loading method with maximum loading capacities of 2000 kN, 500 kN, and 300 kN, respectively. Each loading system contains an independent loading frame, and each end of the loading frame is equipped with a piston rod, which is externally connected to the servo valve to produce independent servo loading (Fig. 1a). The positions of the two loading frames (*σ*_1_ and *σ*_3_) remain fixed, while the third loading frame (*σ*_2_) is placed on a horizontal guide rail and is able to slide along the guide rail, thus facilitating operation and placement of the sample. Each of the three loading frames is orthogonal to the others, and in combination, all form the x, y, z three-dimensional loading space for the test specimen (Fig. 1a and c). The other end of the loading frames (*σ*_2_ and *σ*_3_) are installed with reaction screws, which can be adjusted in length to adapt to different specimen sizes. The true triaxial cell is arranged in the *σ*_2_ loading frame and horizontally connected with both the piston rod and the reaction screw through the loading block (Fig. 1b). After the specimen is placed in the pressure cell, the *σ*_2_ loading frame is slid along the guide rail to the interior of the *σ*_1_ loading frame, where its position is adjusted to place the test specimen directly below the *σ*_1_ piston rod. Figure 1c shows the installation and adjustment of the test system, and the true triaxial cell is in the *σ*_1_, *σ*_2_, and *σ*_3_ 3D loading space.

**Figure 2:**
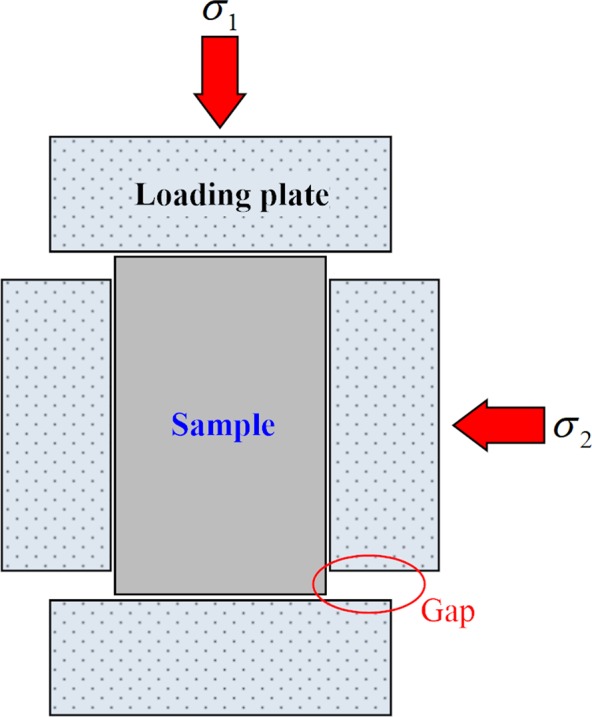
Structure of the traditional true triaxial cell with gaps.

**Figure 3:**
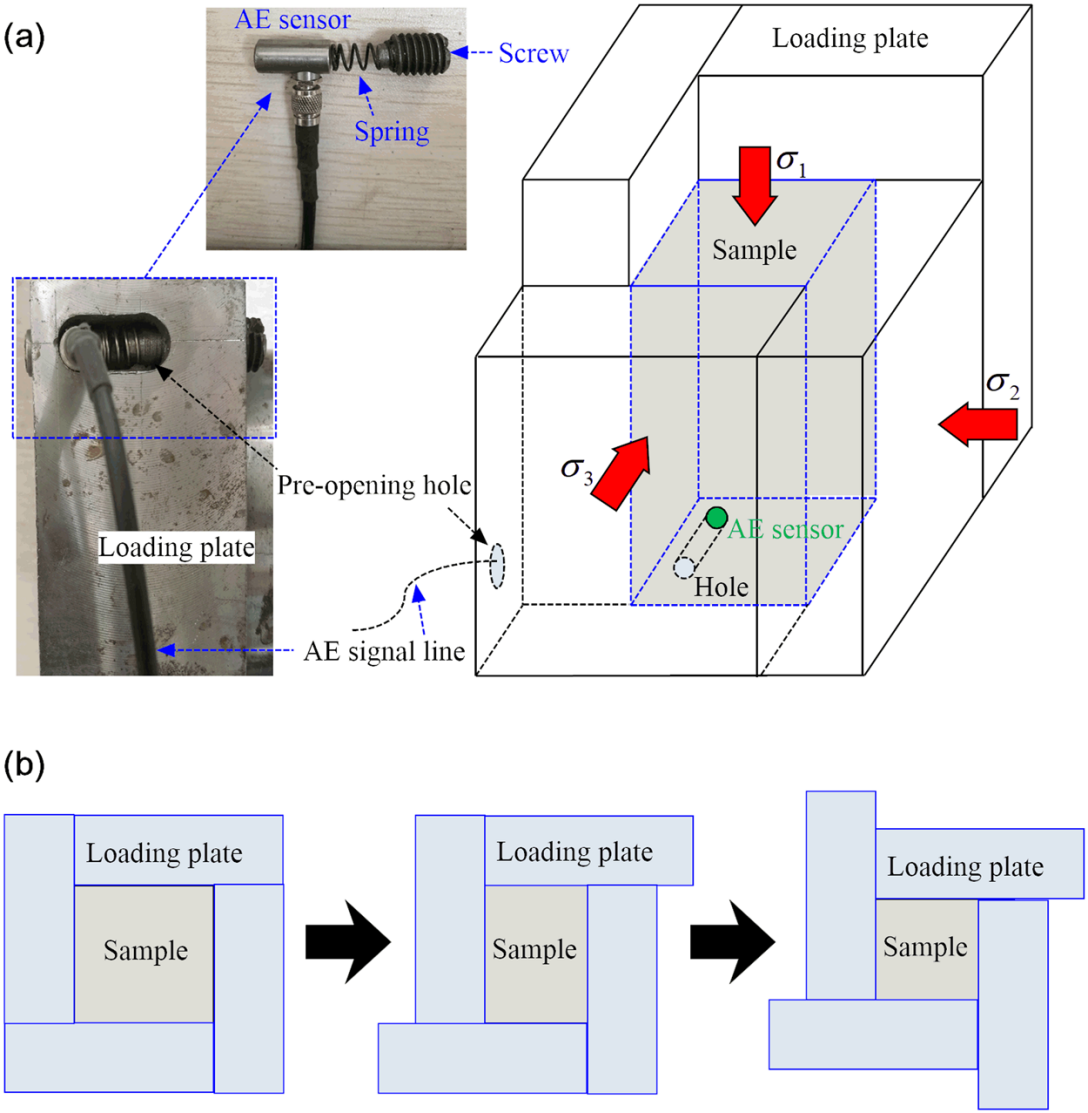
(**a**) Rotating interlocking plate structure in the true triaxial cell and arrangement of AE sensor; (**b**) loading plates synchronously move with the specimen.

The specimen used in this study is a cuboid, 50 × 50 × 100 mm in size. It is placed in a cubic cell that is composed of a rigid structure, which consists of a box-shaped body and loading plates. The true triaxial cell used in this study adopted a fully rigid structure, which is common at present; however, it is easy to squeeze the sample between the loading plates during the loading deformation. To solve this problem, the traditional true triaxial cell typically adopts a design method in which the vertical loading plate is smaller than the sample size, thus leaving a gap between the vertical and the horizontal plate (Fig. 2). However, this gap causes inhomogeneous stress and strain during the test, leading to a decrease of the peak strength and an abnormal failure pattern^22^, which has been defined in this study as the “gap disturbance effect”. To overcome this limitation, the true triaxial cell in this study uses a rotary interlocking platen design, i.e., the loading plates on six surfaces are arranged in a misplaced manner (see Fig. 3a). When the specimen undergoes large deformation, the loading plate pushes the edge movement of the adjacent plate (Fig. 3b), so that the loading plates synchronously move while completely covering the specimen, thus avoiding the “gap disturbance effect”.

During tests, relative displacement between the loading plate and specimen occurs. Friction on the contact surface exerts a negative impact on the test results. To reduce this friction, a lubricant with thickness of 0.5 mm is added onto the contact surface. This lubricant is composed of stearic acid and vaseline at a ratio of 1:1.

A spoke type pressure sensor with a precision of 0.01 kN was installed between the piston rod and the loading block. A rope type displacement sensor with an accuracy of 0.002 mm and a measurement range of 1,000 mm was fixed to the piston rod and reaction force screw. A DS2-8B full information acoustic emission signal analyzer was used to measure the AE activity during tests. The type of AE sensor was a RS-54A with a diameter of 8 mm and a length of 16 mm, which was installed in the loading plate (an 8 mm diameter circular hole was set in the side of the plate). As shown in Fig. 3(a), the AE sensor was pushed into the circular hole through the pre-opening hole and the AE signal line was drawn out from the pre-opening hole, so that the AE sensor was ultimately embedded in the circular hole in direct contact with the sample. The tail of the AE sensor was fixed in the loading plate via spring and screw. Therefore, the AE sensor can be in full contact with the sample surface, thus improving the accuracy of the acoustic emission acquisition signal.

### Specimen preparation

**Figure 4:**
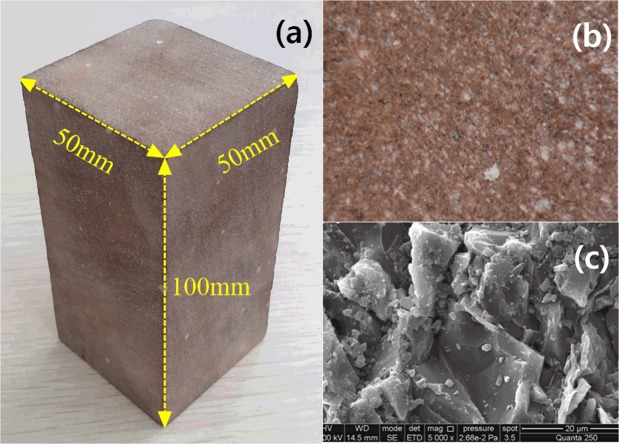
Red sandstone sample: (**a**) Sample size; (**b**) optical microscopic features and (**c**) SEM features of the sandstone sample.

Sandstone was selected for examination in this study. The specimen is composed of feldspar and quartz, as well as a small amount of montmorillonite and hematite ore. This sandstone sample is of medium grain structure with a grain size of 0.1–0.35 mm and a relatively dense mass structure. Grains are relatively uniform with macroscopic uniformity (Fig. 4) and an average density of 2380 kg/m^3^. The specimen was processed according to the rock mechanics test procedure requirements and the end face of the specimen was carefully polished.

### Testing procedure

**Figure 5:**
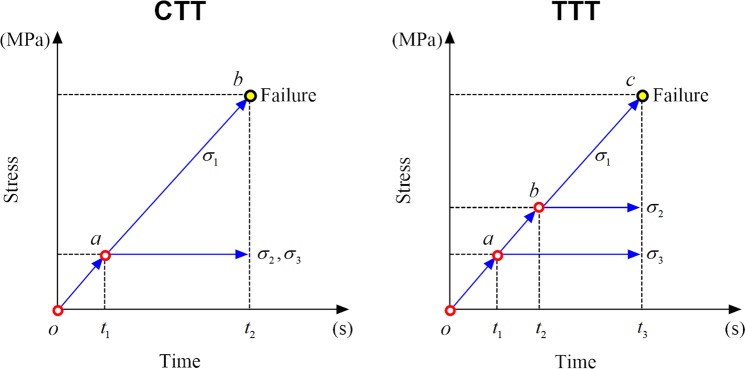
Two different loading programs.

To study the deformation characteristics in the three principal stress directions of the sandstone specimen under true triaxial compression, the differences between conventional triaxial compression tests were comparatively analyzed. Furthermore, the initiation, propagation, and coalescence of interior micro-cracks until failure were evaluated in combination with AE. Therefore, the testing program in this study was designed (Fig. 5) for both the conventional triaxial test (CTT) and the true triaxial test (TTT), respectively.

**CTT**: (1) The sample was subjected to a hydrostatic pressure state (point *a*, *σ*_1_ = *σ*_2_ = *σ*_3_) at a loading rate of 0.2 MPa/s in accordance with the force loading control mode. (2) Under constant confining pressure, axial stress was applied to the specimen at a loading rate of 0.002 mm/s in displacement loading control mode, until specimen failure (*ab*).

**TTT**: (1) The sample was subjected to a hydrostatic pressure state, (point *a*, *σ*_1_ = *σ*_2_ = *σ*_3_) at a loading rate of 0.2 MPa/s in accordance with the force loading control mode (set value of *σ*_3_). (2) Under constant *σ*_3_, the values of *σ*_1_ and *σ*_2_ continuously increased to the set value of *σ*_2_ (*ab*) at identical loading control mode and rate. (3) With constant *σ*_2_ and *σ*_3_, axial stress was applied to the specimen at a loading rate of 0.002 mm/s with the displacement loading control mode, until specimen failure (*bc*).

## Deformation behavior during progressive damage of rock

**Figure 6:**
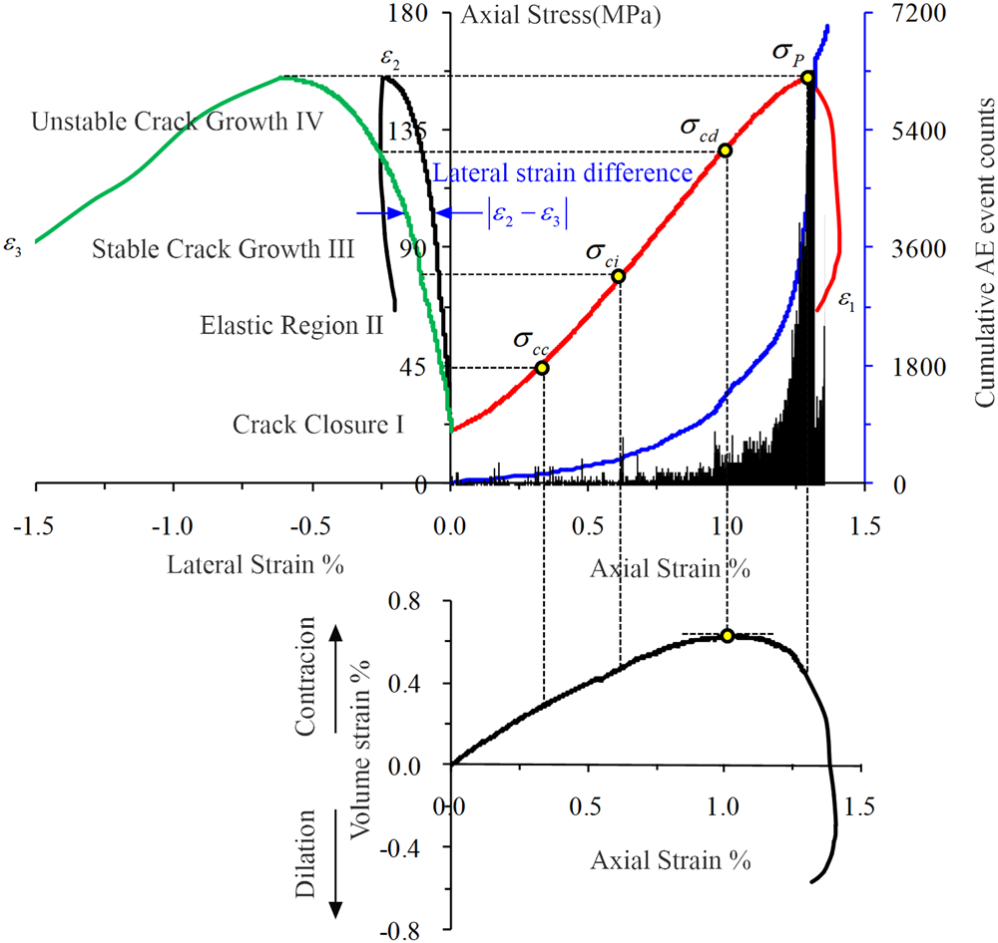
Stress-strain diagram of rock, showing the stages of crack development (*σ*_3_ = 10 MPa, *σ*_2_ = 30 MPa).

Figure 6 shows a typical stress-strain curve for rock tests (*σ*_3_ = 10 MPa, *σ*_2_ = 30 MPa). The deformation properties of rock are closely related to the growth and development of internal cracks in rock. A deep study of the process of rock brittle failure was conducted by Martin using the test^23^. The analysis showed that the failure process is associated with the closure, fracture initiation, expansion, and interactive perforation of cracks. The progressive failure process can be divided into the following stages: (1) the micro crack closure compaction stage; (2) the elastic deformation stage; (3) the stable crack propagation stage; (4) the unstable crack propagation stage; (5) the post-peaking stage.

*σ*_*ci*_ and *σ*_*cd*_ are two important stress values for the characterization of rock mechanical properties. The corresponding strain values *ε*_*ci*_ and *ε*_*cd*_ are key mechanical parameters in the process of rock failure. The stress values (*σ*_*ci*_ and *σ*_*cd*_) and strain values (*ε*_*ci*_ and *ε*_*cd*_) play an important guiding role for the study of rock deformation, strength, and other specific mechanical properties. The interpretation of *σ*_*cd*_ is more objective, which is defined as the stress value that corresponds to the volume strain inflection point, i.e., the applied axial stress corresponds to the maximum volume strain. This method was used to determine the damage stress of the rock. The recognition of *σ*_*ci*_ was relatively difficult, and two types of methods are currently available^24,25^: the strain method and the acoustic emission method. Eberhardt *et al*.^26^ pointed out that the crack initiation stress corresponds to the stress during the first AE signal with an obvious increase. In this study, the AE method was adopted to determine *σ*_*ci*_. Considering that the AE ringing counts are more sensitive than AE event counts as well as more vulnerable to outside influence, the AE event count method was adopted to determine the crack initiation stress of the sandstone specimen.

**Figure 7:**
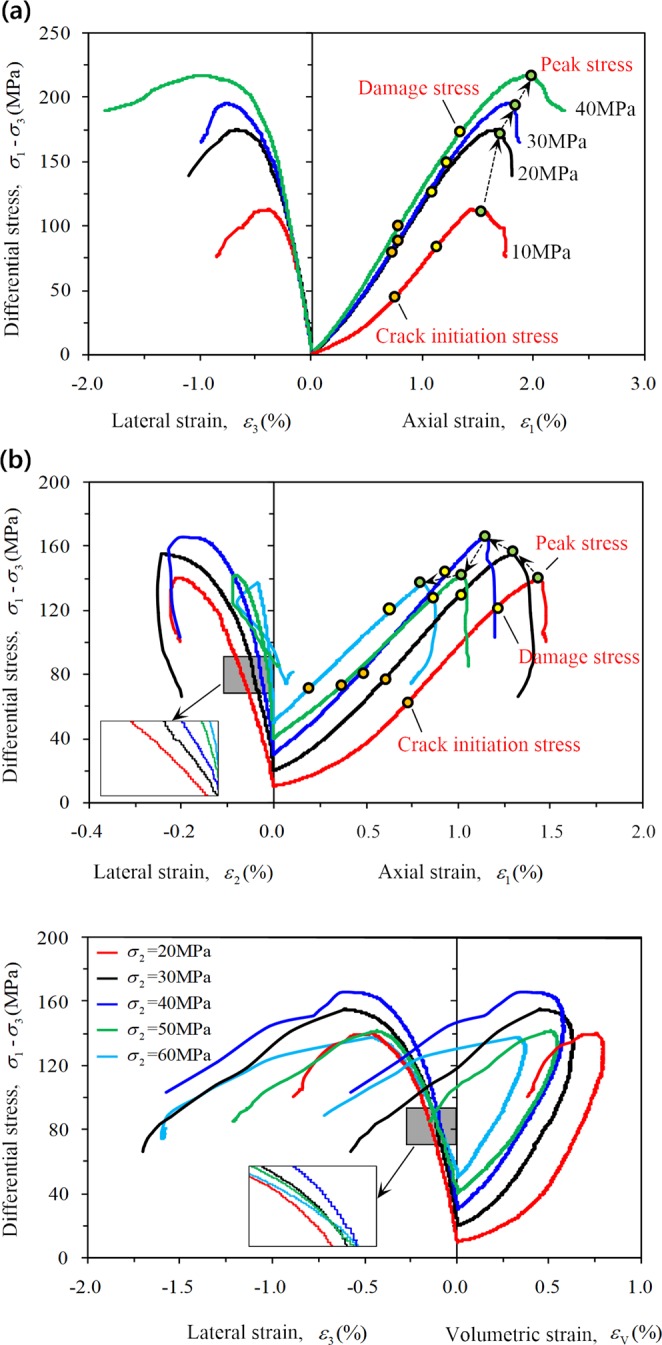
Stress-strain curves for sandstone: (**a**) CTT; (**b**) TTT (same color of the curve represents the same stress state).

Figure 7 shows stress-strain curves and crack initiation stress (*σ*_*ci*_), damage stress (*σ*_*cd*_), and peak stress (*σ*_*p*_) of rock with various confining pressures under CTT and TTT conditions. The rock stress-strain characteristics for four confining pressures under CTT conditions are similar (Fig. 7a). The axial strain, lateral strain, and peak strength of the rock all increase with increasing confining pressure, which shows that the increasing confining pressure improves the ability of the rock to withstand deformation in the both the axial and lateral directions.

Figure 7 (b) show the relationships between the differential stress (*σ*_1_–*σ*_3_) and principal strain (*ε*_1_, *ε*_2_, and *ε*_3_) as well as the volumetric strain (*ε*_V_) under true triaxial compression. Since different intermediate principal stresses under true triaxial compression were utilized, the test process could be divided into one section with a loading control by force (*o* → *a* → *b*) and another section with a loading control by displacement (*b* → *c*). To facilitate the analysis, the stress-strain characteristics of sandstone were only analyzed for the loading control of the displacement section (*b* → *c*).

During the initial compaction stage, the rock deformation is not obvious due to the effect of loading control by force (*o* → *a* → *b*). The rock rapidly enters the elastic stage in which the axial curve is linear; with increasing *σ*_2_, the strain of the rock gradually decreased during loading control by displacement (*b* → *c*), and its lateral expansion gradually plays a more dominant role.

The strain curve in the direction of *σ*_2_ gradually steepens with increasing *σ*_2_, indicating that the deformation (expansion) in this direction is limited with increasing *σ*_2_. After the peak stress is reached, the rock is subjected to compression in the *σ*_2_ direction, especially for *σ*_2_ of 50 MPa or 60 MPa, indicating that severe brittle failure of the rock occurs and the rock extends rapidly along the *σ*_3_ direction.

**Figure 8:**
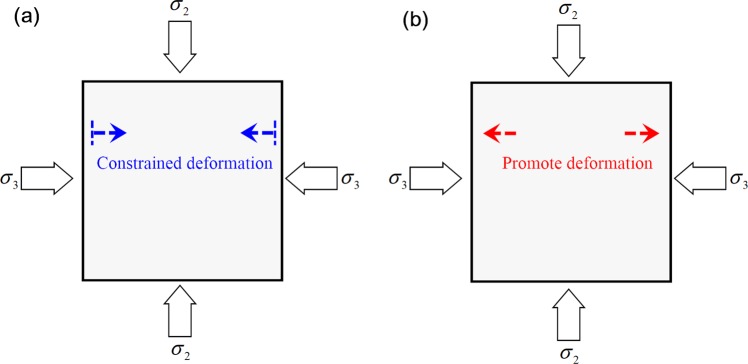
Effect of different intermediate principal stresses on the rock in direction of the minimum principal stress: (**a**) constrained deformation; (**b**) promoted deformation.

The lateral expansion of rock mainly occurs along the direction of minimum principal stress, and the curve first steepens and then slows down with increasing *σ*_2_. This increasing *σ*_2_ can promote the rock to expand in the direction of *σ*_3_ on the one hand, while on the other hand, *σ*_2_, as confining pressure, can limit the lateral expansion of the rock specimen. The amplification area results showed that: when *σ*_2_ was 20 MPa, 30 MPa, or 40 MPa (Fig. 8a), the increasing *σ*_2_ played a protective role for the rock and limited deformation in the direction of *σ*_3_ (lateral restraint effect). When *σ*_2_ was 50 MPa or 60 MPa (Fig. 8b), the increasing *σ*_2_ damaged the rock and accelerated the deformation in the direction of *σ*_3_ (which is an effect of the damage).

**Figure 9:**
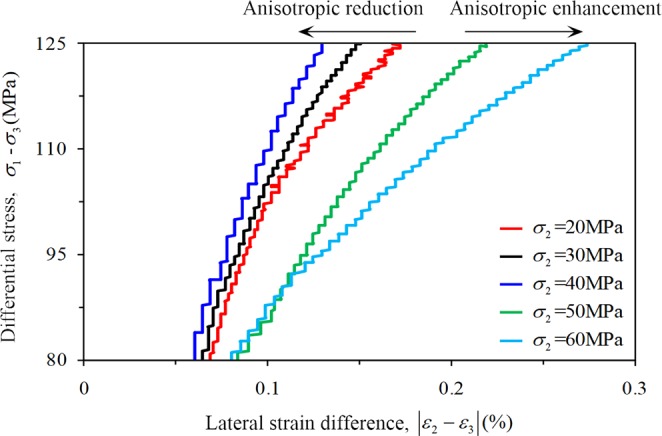
Evolution law of lateral strain difference (TTT).

To compare the influence of *σ*_2_ on the anisotropic characteristics of rock, this study defines the lateral strain difference |*ε*_2_ – *ε*_3_| as the difference (absolute value) between the intermediate principal strain and the minimum principal strain (Fig. 6). The magnitude of the difference |*ε*_2_ − *ε*_3_| can reflect the anisotropic characteristics of rocks, and a larger difference indicates more obvious anisotropic characteristics. When *σ*_2_ was 20 MPa, 30 MPa, and 40 MPa, the difference |*ε*_2_ – *ε*_3_| gradually decreased with increasing *σ*_2_, indicating that the anisotropic characteristics gradually weakened. While *σ*_2_ was 50 MPa and 60 MPa, the difference |*ε*_2_ – *ε*_3_| increased noticeably, which indicated that the characteristics of rock anisotropic improved significantly (Fig. 9).

The *ε*_V_ of rock first increased and then decreased, indicating that the specimen was gradually compressed and begins to dilate. The inflection point of *ε*_V_ is the point where the rock volume stops to be compressed and begins to expand. The inflection point of the volume strain is more easily reached with increasing *σ*_2_.

**Figure 10:**
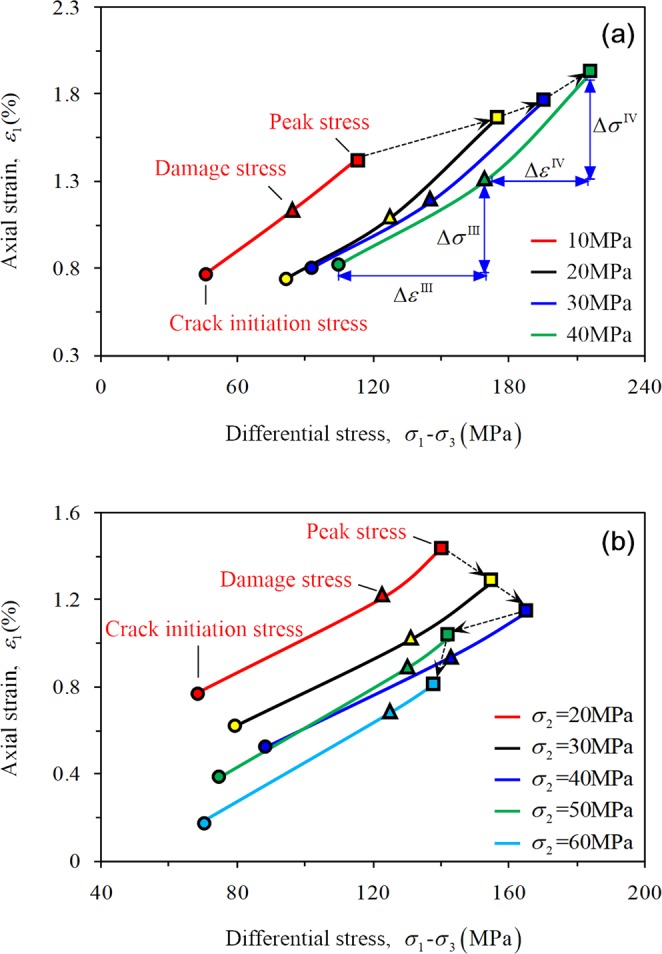
Evolution law of deformation and strength of sandstone in progressive failure: (**a**) CTT; (**b**) TTT.

Figure 10 shows the changing patterns of progressive failure of sandstone under both CTT and TTT conditions. The variation rule of crack initiation stress (*σ*_*ci*_), damage stress (*σ*_*cd*_), and peak intensity (*σ*_*p*_) as well as the corresponding strain of the rock under CTT conditions increased with increasing confining pressure (Fig. 10a). Because there are microcracks in the rock, by improving the confining pressure, the development of cracks can be effectively prevented, and the expansion of internal cracks of the rock can be laterally restrained.

Variations of *σ*_*ci*_, *σ*_*cd*_ and *σ*_*p*_ and the corresponding strain under TTT conditions differ in rocks under CTT conditions. All increase first and then decrease with maximum stress occurring at *σ*_2_ = 40 MPa (Fig. 10b). When *σ*_2_ is small, the slippage deformation of internal micro cracks is restrained with increasing *σ*_2_. However, when *σ*_2_ exceeds 40 MPa, the lateral constraint gradually evolves to the damage effect of rock, which promotes crack expansion.

In this study, the stress and strain difference values are respectively defined as the value of the differential stress and strain change during the stable crack propagation stage III and the unstable crack propagation stage IV:

1 $$\{\begin{array}{rcl}{\rm{\Delta }}{\varepsilon }^{{\rm{III}}} & = & {\varepsilon }_{cd}-{\varepsilon }_{ci}\\ {\rm{\Delta }}{\sigma }^{{\rm{III}}} & = & {\sigma }_{cd}-{\sigma }_{ci}\\ {\rm{\Delta }}{\varepsilon }^{{\rm{IV}}} & = & {\varepsilon }_{p}-{\varepsilon }_{cd}\\ {\rm{\Delta }}{\sigma }^{{\rm{IV}}} & = & {\sigma }_{p}-{\sigma }_{cd}\end{array}$$

The internal crack propagation during stage III remains stable and the rock stress and strain difference values Δ*σ*^III^ and Δ*ε*^III^ between CTT and TTT conditions are close. However, during the unstable crack growth stage (IV), the rock stress and strain difference values Δ*σ*^IV^ and Δ*ε*^IV^ under the TTT condition (especially when *σ*_2_ is 50 MPa or 60 MPa) is significantly smaller than under the CTT condition. This indicates that the internal crack propagation of the rock rapidly increases due to *σ*_2_ > *σ*_3_, and the rock reached peak strength with a lower stress and strain difference value.

### Micro crack closure compaction stage (I)

Stage I is the micro crack closure compaction stage of the rock. The existence of this stage depends on the density and geometry of existing cracks. The stress threshold during this stage is the crack closure stress (*σ*_*cc*_). Once internal cracks are completely closed, the specimen enters the elastic deformation stage (Stage II). In this study, the existing initial cracks are partially or completely closed with the force loading control mode. As a result, the initial compression stage of the rock is not obvious and the rock quickly enters the elastic deformation stage.

### Elastic deformation stage (II)

During the elastic deformation stage, the axial and lateral curves show good linear characteristics under each confining pressure, and the confining pressure significantly affects the degree of steepness of the axial curve. A higher confining pressure indicates a steeper curve under CTT conditions (Fig. 7a), which further indicates that the Young’s modulus of red sandstone increases with increasing confining pressure. It first increases and then decreases with increasing *σ*_2_ under TTT conditions (Fig. 7b). The degree of the lateral curve steepness was not particularly obvious under both conditions, which indicates that the effect of the confining pressure on the lateral curve steepness is very small for sandstone.

### Stable crack growth stage (III)

**Figure 11:**
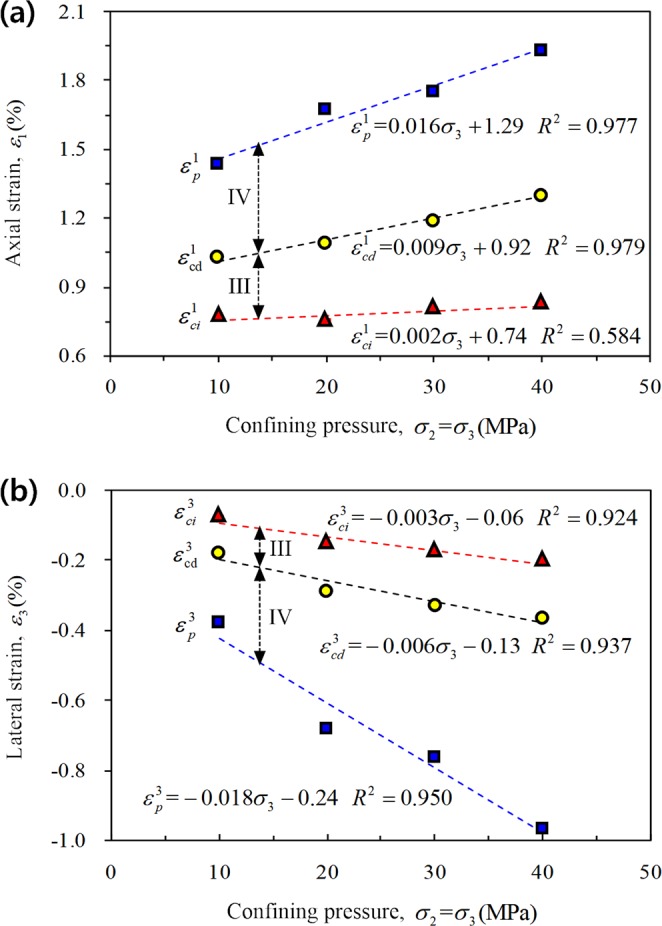
Influence of confining pressure on strain corresponding to each characteristic stress under CTT conditions: (**a**) Axial strain and (**b**) lateral strain.

**Figure 12:**
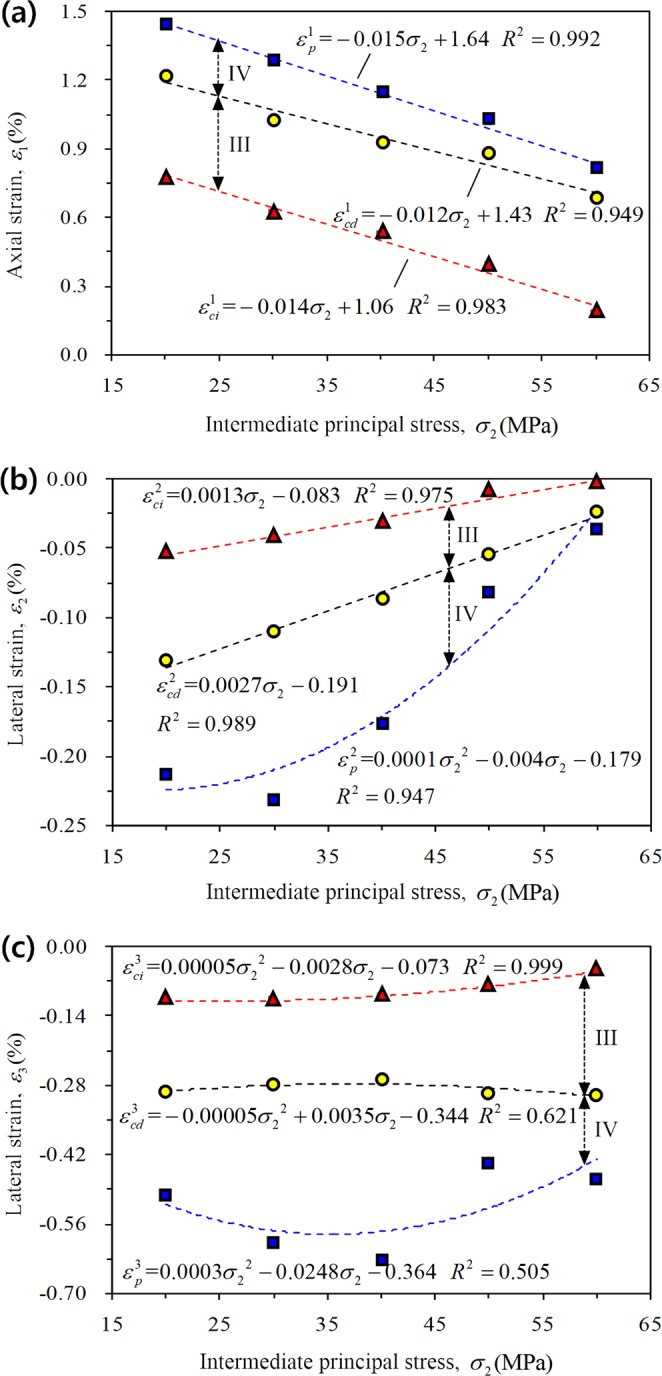
Influence of the intermediate principal stress on the strain corresponding to each characteristic stress under TTT conditions: (**a**) Axial strain *ε*_1_, (**b**) lateral strain *ε*_2_, and (**c**) lateral strain *ε*_3_.

Figures 11 and 12 show the strain characteristics of progressive damage in rock in different stages under CTT and TTT conditions, respectively, and the fitting relation of strain increments in stable crack growth stage (III) are as follows:

① CTT:

2 $$\{\begin{array}{l}{\rm{\Delta }}{\varepsilon }_{1}={\varepsilon }_{cd}^{1}-{\varepsilon }_{ci}^{1}=0.007{\sigma }_{3}+0.18\\ {\rm{\Delta }}{\varepsilon }_{3}={\varepsilon }_{cd}^{3}-{\varepsilon }_{ci}^{3}=-\,0.003{\sigma }_{3}-0.07\end{array}$$

3 $${\rm{\Delta }}{\varepsilon }_{{\rm{v}}}={\rm{\Delta }}{\varepsilon }_{1}+2{\rm{\Delta }}{\varepsilon }_{3}=0.001{\sigma }_{3}+0.04$$

② TTT:

4 $$\{\begin{array}{l}{\rm{\Delta }}{\varepsilon }_{1}={\varepsilon }_{cd}^{1}-{\varepsilon }_{ci}^{1}=0.002{\sigma }_{2}+0.37\\ {\rm{\Delta }}{\varepsilon }_{2}={\varepsilon }_{cd}^{2}-{\varepsilon }_{ci}^{2}=0.0014{\sigma }_{2}-0.108=-\,(0.108-0.0014{\sigma }_{2})\\ {\rm{\Delta }}{\varepsilon }_{3}={\varepsilon }_{cd}^{3}-{\varepsilon }_{ci}^{3}=-\,0.0001{{\sigma }_{2}}^{2}+0.0063{\sigma }_{2}-0.271\end{array}$$

5 $${\rm{\Delta }}{\varepsilon }_{{\rm{v}}}={\rm{\Delta }}{\varepsilon }_{1}+{\rm{\Delta }}{\varepsilon }_{2}+{\rm{\Delta }}{\varepsilon }_{3}=-\,0.0001{{\sigma }_{2}}^{2}+0.0099{\sigma }_{2}-0.009$$

**Figure 13:**
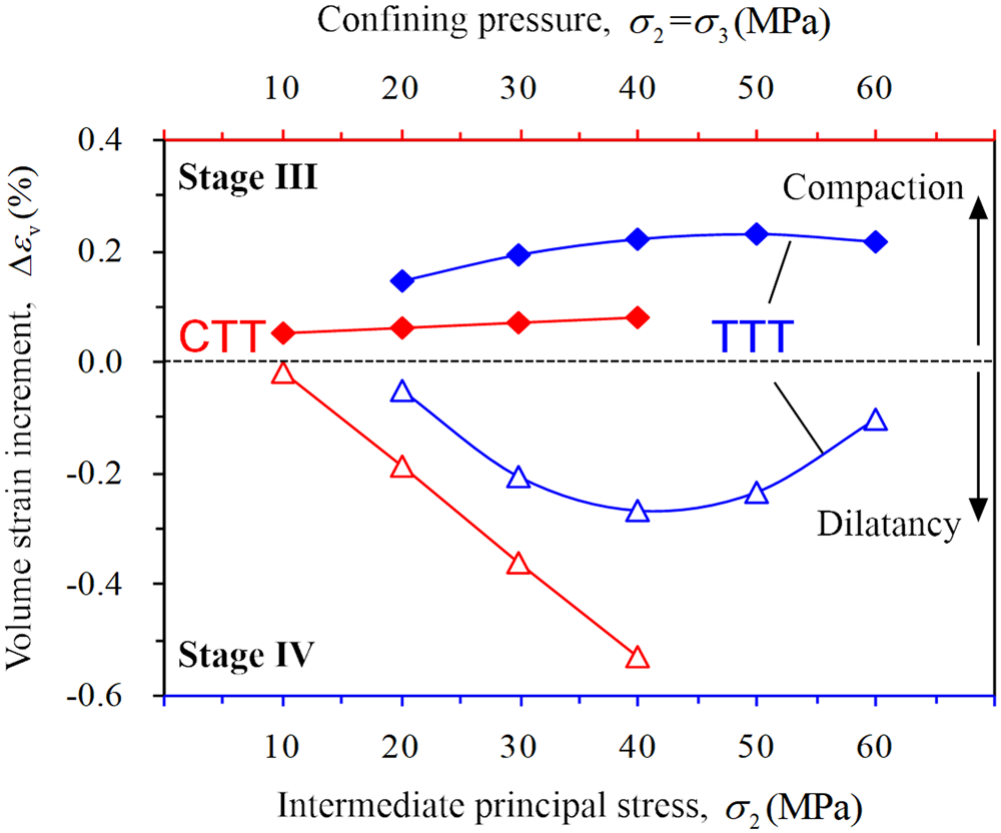
Variation regularity of volume strain increment for different stages of rock.

Clearly, under CTT conditions, the axial strain (Fig. 11a) and the lateral strain (Fig. 11b) during different stages basically follow a linear increase with increasing confining pressure. The axial strain increment of the rock exceeds the lateral strain increment as shown by Eq. 2. The rock at the stable crack growth stage is mainly compressed (Eq. 3), and with increasing confining pressure, the volume strain increment increases slightly (Fig. 13).

The stress–strain characteristics of rock were only analyzed for the loading control by displacement section under TTT conditions. At different stages, both the axial strain (Fig. 12a) and the lateral strain *ε*_2_ (expansion is negative, Fig. 12b) decrease with increasing *σ*_2_, and the lateral strain *ε*_3_ shows a stronger fluctuation (Fig. 12c). During stage III, the axial strain increment increases gradually and the lateral strain increment Δ*ε*_2_ decreases (expansion is negative); however, the lateral strain increment Δ*ε*_3_ of the rock specimen first decreases and then increases with increasing *σ*_2_ (Eq. 4). Equation 5 can be obtained and the rock is also mainly compressed at this stage. With increasing *σ*_2_, the volume strain increment first shows an increase and then follows a decreasing trend (Fig. 13).

**Figure 14:**
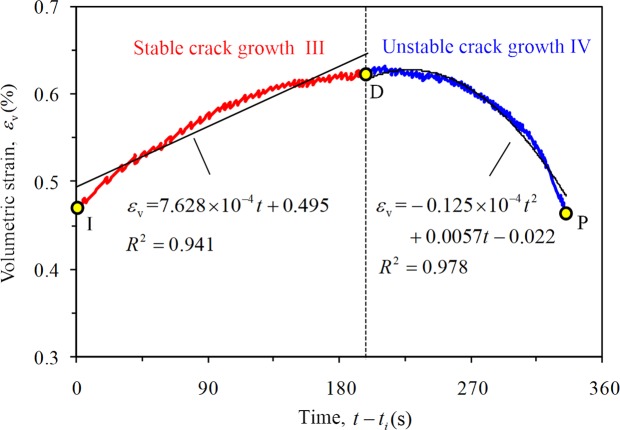
Variation law of volume strain with time and its fitting curve at stable and unstable stage (*σ*_3_ = 10 MPa, *σ*_2_ = 30 MPa); *t*_*i*_ represents the moment of rock crack initiation.

Figure 14 shows the variation in volume strain of rock with time and the corresponding fitting curve. The volume strain approximately exhibits a linear relationship with the time in stable crack growth stage, and ∂*ε*_v_/∂*t* is defined as the volume strain rate where the compression is positive and the expansion is negative. The fitting relation indicates that the volume strain rate of the rock is constant at this stage.

**Figure 15:**
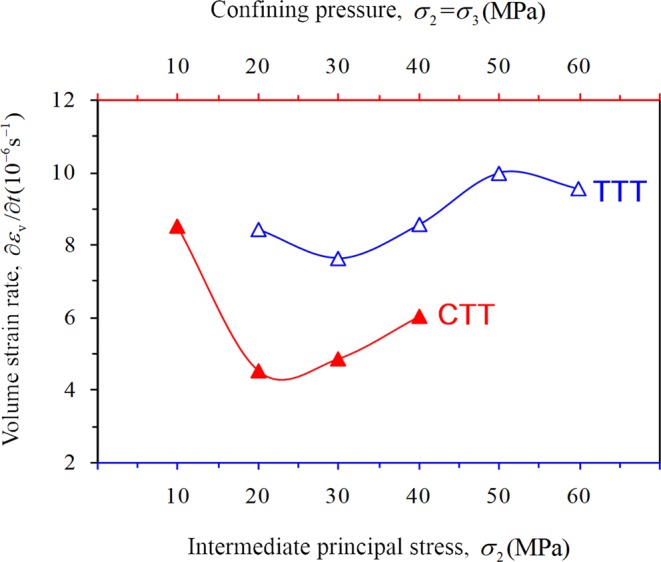
Variation regularity of volume strain rate of rock at the stable stage.

Figure 15 shows the variation regularity of the volume strain rate of rock with confining pressure under CTT and TTT conditions, respectively. The overall growth trend of the volume strain rate (compression) of rock under CTT conditions (except for confining pressure of 10 MPa) indicates that the axial strain rate exceeds the lateral expansion rate, and the effect of confining pressure is more obvious: a greater confining pressure indicates a higher volume strain rate, i.e., a stronger binding of the rock. The true triaxial volume strain rate of rock first increases and then decreases (except for *σ*_2_ = 20 MPa), which indicates that the confining pressure is obvious when *σ*_2_ is small, and the lateral expansion rate of rock is low. When *σ*_2_ exceeds 50 MPa, the lateral swelling of the rock increases significantly, resulting in a decreasing trend of the volume strain rate (compression).

**Figure 16:**
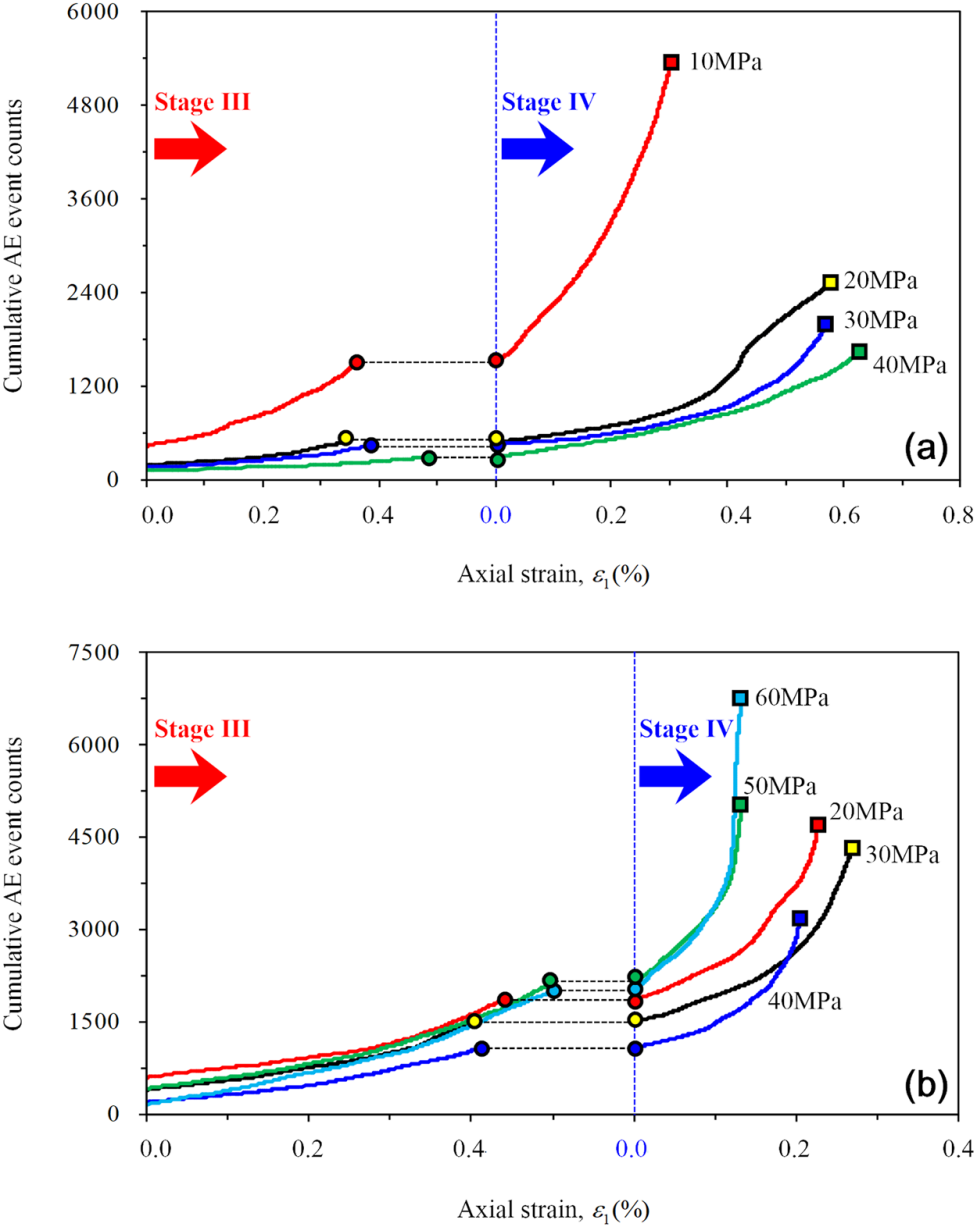
Typical curve of cumulative acoustic AE counts with strain change in stage III and IV: (**a**) CTT and (**b**) TTT.

In this study, the development of micro-cracks in the rock specimen was detected via AE. At the stable crack growth stage, internal micro cracks in the rock gradually generate and AE counts gradually increase. Under CTT conditions, the AE activity follows a “gradually decreasing” trend with increasing confining pressure (Fig. 16a), which indicates that the increase of confining pressure causes gradual inhibition of the crack growth. The AE activity of the specimen first decreases and then increases with increasing *σ*_2_ under TTT conditions (Fig. 16b). Due to the stable extension of the crack inside the rock at this stage, when *σ*_2_ is 20, 30, and 40 MPa, the expansion of internal cracks is laterally constrained by the confining pressure, thus causing the lateral expansion strain to decrease slightly during this stage. When *σ*_2_ is 50 or 60 MPa, the expansion of micro cracks is gradually promoted and the fracture development accelerates, causing the lateral expansion to increase at this stage. This also explains the reason why the volume strain rate of rock first increases and then decreases under TTT conditions.

### Unstable crack growth stage (IV)

When the axial stress gradually increases toward the damage stress, the rock enters the unstable crack stage (IV). And the fitting relationships of strain increments in this stage are as follows:

① CTT:

6 $$\{\begin{array}{l}{\rm{\Delta }}{\varepsilon }_{1}={\varepsilon }_{p}^{1}-{\varepsilon }_{cd}^{1}=0.007{\sigma }_{3}+0.37\\ {\rm{\Delta }}{\varepsilon }_{3}={\varepsilon }_{p}^{3}-{\varepsilon }_{cd}^{3}=-\,0.012{\sigma }_{3}-0.11\end{array}$$

7 $${\rm{\Delta }}{\varepsilon }_{{\rm{v}}}={\rm{\Delta }}{\varepsilon }_{1}+2{\rm{\Delta }}{\varepsilon }_{3}=-\,0.017{\sigma }_{3}+0.15$$

② TTT:

8 $$\{\begin{array}{l}{\rm{\Delta }}{\varepsilon }_{1}={\varepsilon }_{p}^{1}-{\varepsilon }_{cd}^{1}=-\,0.003{\sigma }_{2}+0.21\\ {\rm{\Delta }}{\varepsilon }_{2}={\varepsilon }_{p}^{2}-{\varepsilon }_{cd}^{2}=0.0001{\sigma }_{2}^{2}-0.0067{\sigma }_{2}-0.012\\ {\rm{\Delta }}{\varepsilon }_{3}={\varepsilon }_{p}^{3}-{\varepsilon }_{cd}^{3}=0.00035{\sigma }_{2}^{2}-0.0283{\sigma }_{2}-0.02\end{array}$$

9 $${\rm{\Delta }}{\varepsilon }_{{\rm{v}}}={\rm{\Delta }}{\varepsilon }_{1}+{\rm{\Delta }}{\varepsilon }_{2}+{\rm{\Delta }}{\varepsilon }_{3}=0.00045{\sigma }_{2}^{2}-0.038{\sigma }_{2}-0.001$$

The lateral strain increment of the rock exceeds the axial strain increment under CTT conditions (Eq. 6), indicating that the lateral strain gradually dominates and the allowable lateral expansion value of rock increases. The rock mainly expands during this stage (Eq. 7), and the volume strain increment exhibits a sharp increase with increasing confining pressure (Fig. 13). On the other hand, the bearing capacity of rock toward lateral expansion enhances with increasing confining pressure.

Both the axial strain increment Δ*ε*_1_ (compression) and the lateral strain increment Δ*ε*_2_ (expansion) decrease substantially with increasing *σ*_2_ under TTT conditions (Eq. 8), while Δ*ε*_3_ first increases and then decreases. When *σ*_2_ is 50 and 60 MPa, the three values decrease rapidly, indicating that the rock reaches the peak strength within a short time. The volume strain increment (dilation) first increases and then decreases with increasing *σ*_2_ (Fig. 13).

According to the fitting curve between the volume strain of rock and the time (Fig. 14), the binomial relation between the rock volume strain and time can be presented as follows:

10 $${\varepsilon }_{{\rm{v}}}=a{(t-{t}_{d})}^{2}+b(t-{t}_{d})+c$$

*t*_*d*_ represents the moment of rock damage stress, *a*, *b*, and *c* are the fitting coefficients, and the volume strain rate is:

11 $$\partial {\varepsilon }_{{\rm{v}}}/\partial t={\rm{2}}a(t-{t}_{d})$$

**Figure 17:**
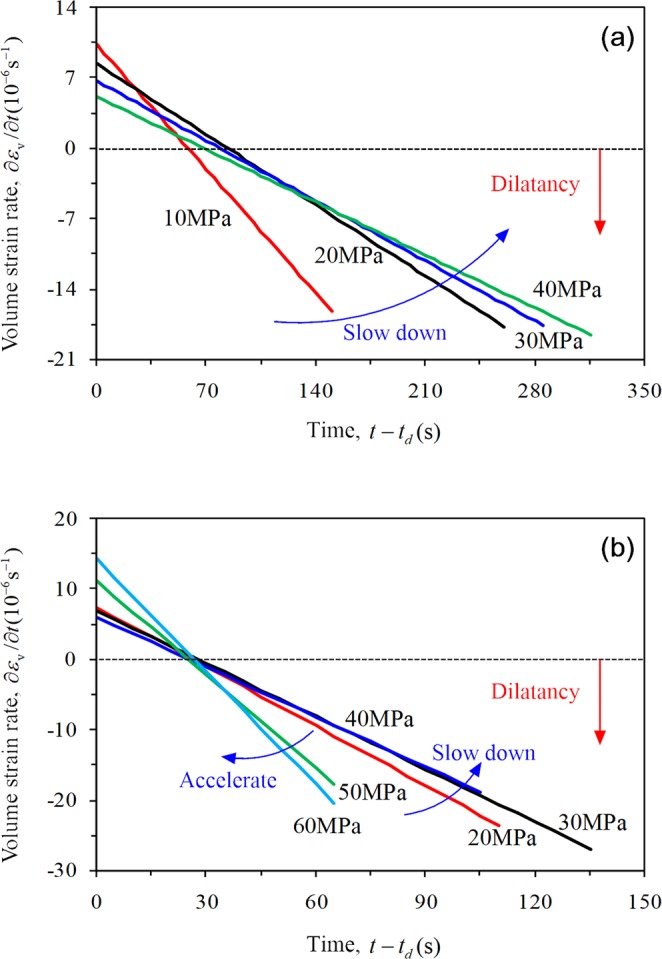
Variation of the volume-strain rate with time at the unstable stage under different confining pressure conditions; *t*_*d*_ represents the moment of rock damage stress. (**a**) CTT and (**b**) TTT.

The volume strain rate (dilation) of rock increases with time during this stage (Eq. 11). Under CTT conditions, the volume expansion rate of rock gradually slows down (Fig. 17a), indicating that the lateral expansion of rock is gradually inhibited. Under TTT conditions, when *σ*_2_ is 20, 30, or 40 MPa, the volume expansion rate of rock gradually decreases. The lateral expansion of rock is gradually inhibited with increasing *σ*_2_. When *σ*_2_ is 50 and 60 MPa, the rock volume rate is obviously accelerated, indicating that the confining pressure gradually promotes swelling deformation of the rock specimen (Fig. 17b).

**Figure 18:**
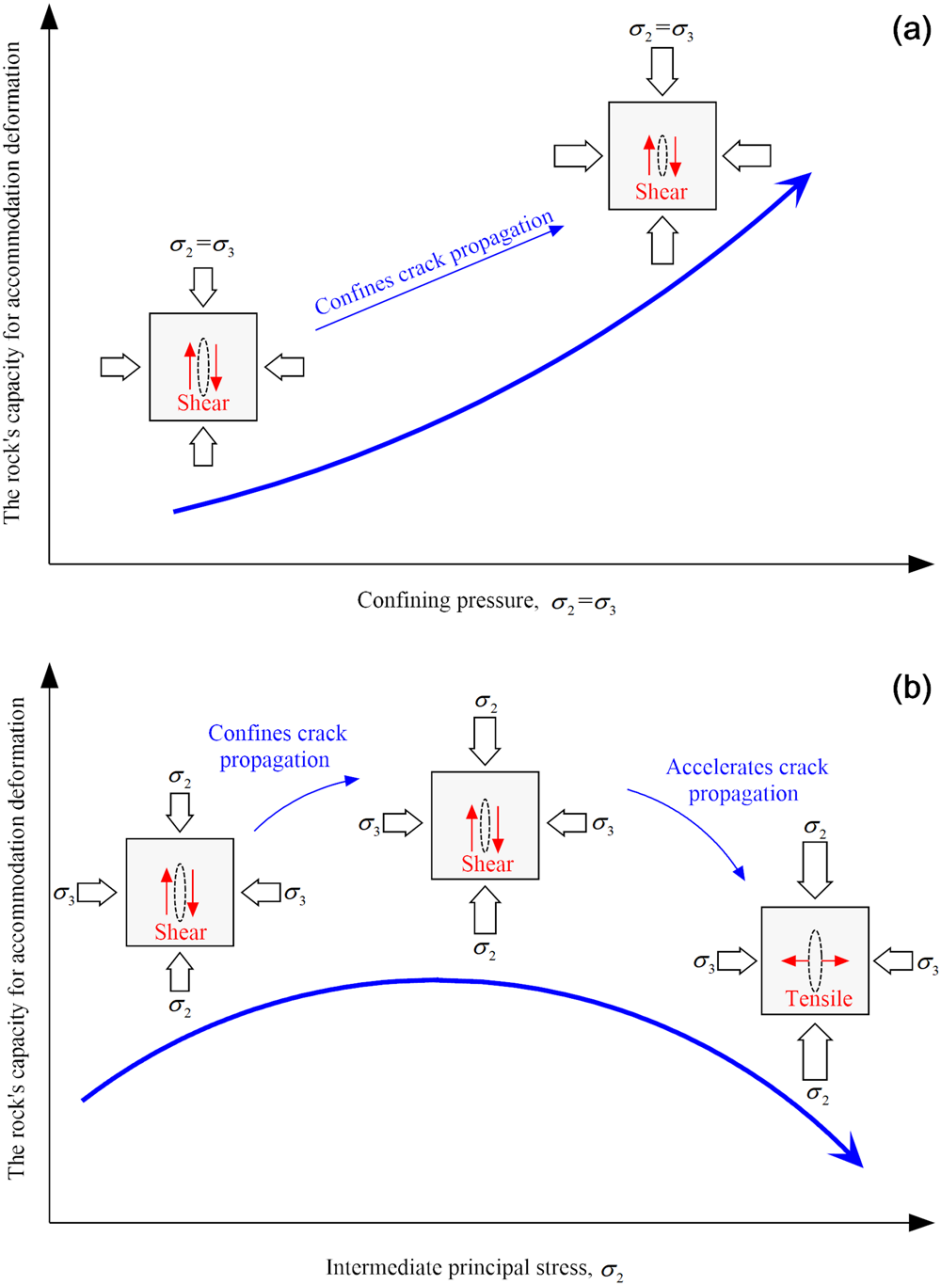
Effect of different confining pressures on the internal cracks of rock and the rock bearing deformation capacity: (**a**) CTT and (**b**) TTT.

At this stage, AE counts sharply increases, and the interactions among cracks intensively increases. Under CTT compression (Fig. 16a), the law of AE activity is similar to that during the stable stage: AE activity gradually declines and the cumulative AE counts gradually decrease when the peak value is reached. The binding effect of the confining pressure renders the generation of internal fractures and the shear slip of cracks to slow, thus yielding comparatively low AE counts. A higher confining pressure indicates a stronger binding effect, which enhances the rock’s capacity to bear lateral expansion (Fig. 18a).

Under TTT conditions (Fig. 16b), when *σ*_2_ is 20, 30, or 40 MPa, the cumulative AE counts decrease with no “abrupt increasing” phenomenon, and more continuous AE are internally produced in the rock. In combination with the AE characteristics under CTT conditions, numerous shear-slip cracks are produced in this stage. Increasing *σ*_2_ can constrain the shear-slip effect among internal cracks; therefore, the rock is protected and the lateral deformation-support capability gradually increases. When *σ*_2_ is 50 and 60 MPa, the cumulative AE counts rapidly increase with an “abrupt increase”, indicating that the lateral deformation *ε*_2_ is inhibited while the lateral expansion *ε*_3_ is greatly promoted, meaning that more stretching types of cracks are produced and perforated. As a result, the failure plane forms rapidly. Therefore, the function of lateral confinement gradually evolves into the damage effect of rock, leading to a strong reduction of the deformation-support capability (Fig. 18b).

## Discussion

**Figure 19:**
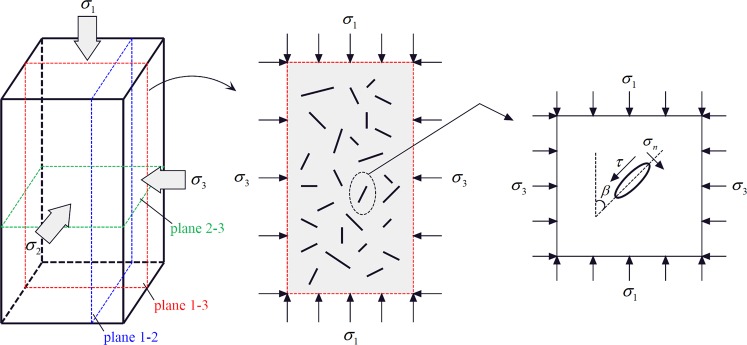
Three-dimensional model with microcrack defects in rock sample under triaxial conditions.

Rock is one of the most common complex geological materials. During the process of diagenesis and geological evolution, defects and damages of different sizes and shapes inevitably form inside the rock. To further analyze the rock deformation mechanism during progressive rock damage, a three-dimensional rock model with microcrack defects under triaxial conditions is established (Fig. 19), and the stress of planes 1–2, 1–3, and 2–3, containing the fracture plane, are studied, respectively (without considering the normal force that is perpendicular to the plane on the micro-crack propagation). Taking plane 1–3 as example, a crack-containing unit is selected, in which the long and short axes of the crack are 2a and 2b, respectively, the dip angle is *β*, and the adjacent microcracks are assumed without mutual influence. The normal stress and tangential stresses on the microcrack surface are:

12 $$\{\begin{array}{rcl}{\sigma }_{n} & = & \frac{1}{2}[({\sigma }_{1}+{\sigma }_{3})+({\sigma }_{1}-{\sigma }_{3})\,\cos \,2\beta ]\\ \tau  & = & \frac{1}{2}({\sigma }_{1}-{\sigma }_{3})\,\sin \,2\beta \end{array}$$

Assuming that the angle between any point on the perimeter of the elliptical crack and the long axis is *α*, the maximum tangential stress must occur near the end of the ellipse with the smallest radius of curvature, i.e. *α* → 0. According to the Inglis solution^27^, the approximate expression of the tangential stress around the crack is:

13 $$\sigma (\alpha )=\frac{2({\sigma }_{n}m+\tau \alpha )}{{m}^{2}+{\alpha }^{2}}$$

Further expansion occurs at the maximum shear stress *σ*(*α*) around the crack:

14 $$\frac{\partial \sigma (\alpha )}{\partial \alpha }=\frac{\partial }{\partial \alpha }\frac{2({\sigma }_{n}m+\tau \alpha )}{{m}^{2}+{\alpha }^{2}}=0$$

Therefore, the extreme value of the tangential stress *σ*(*α*) around the crak can be expressed as:

15 $$\sigma (\alpha )=\frac{1}{m}({\sigma }_{n}\pm \sqrt{{{\sigma }_{n}}^{2}+{\tau }^{2}})$$

The equivalent tangential stress *mσ*(*α*) can be provided by substitution of Eqs (12) and (15):

16 $$m\sigma (\alpha )=\frac{{\sigma }_{1}+{\sigma }_{3}}{2}-\frac{{\sigma }_{1}-{\sigma }_{3}}{2}\,\cos \,2\beta \pm \sqrt{\frac{{\sigma }_{1}^{2}+{\sigma }_{3}^{2}}{2}-\frac{{\sigma }_{1}^{2}-{\sigma }_{3}^{2}}{2}\,\cos \,2\beta }$$

When the equivalent tangential stress *mσ*(*α*) around the crack reaches a critical value [*mσ*(*α*)], the crack propagates and causes rock failure. It should be pointed out that due to the low tensile strength of the rock, the critical value [*mσ*(*α*)] is the local tensile strength of the rock. In rock mechanics, the tensile stress is defined as a negative value; therefore, the extreme value is the negative value with the largest absolute value. The smaller *mσ*(*α*) (absolute value) is, the more difficult it is to expand the internal fissure of rock.

**Figure 20:**
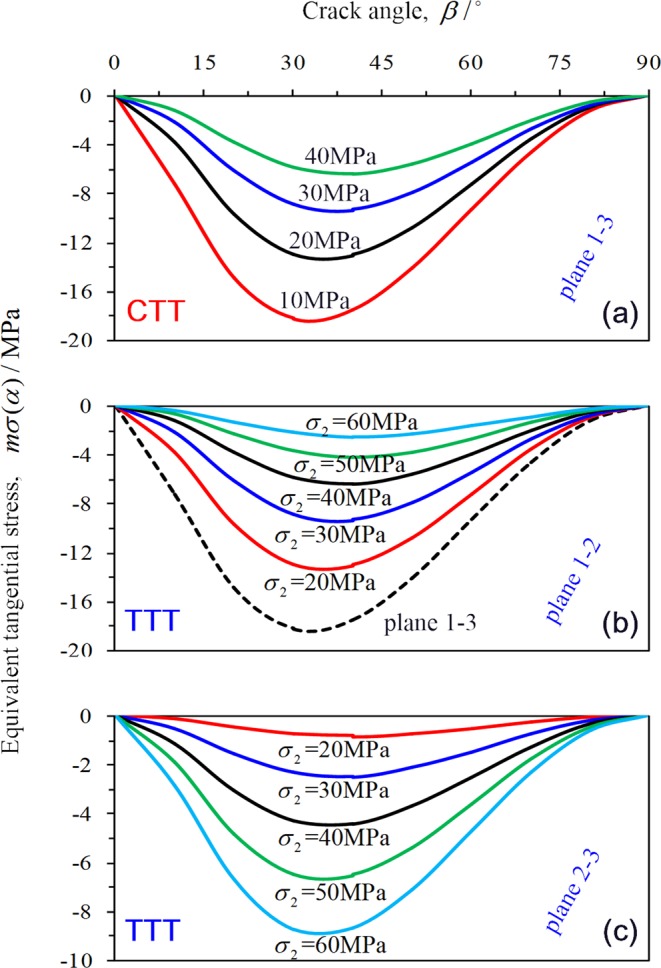
Variation of equivalent tangential stress in different planes.

In this study, the stress *σ*_1_ = 100 MPa, *σ*_2_ = *σ*_3_ = 10–40 MPa is selected under CTT conditions and compared to the variation of different equivalent shear stress *mσ*(*α*) in the plane 1–3 (Fig. 20a). With increasing confining pressure, the equivalent tangential stress *mσ*(*α*) of rock decreases gradually (absolute value), indicating that the internal fracture of rock is gradually suppressed and the lateral deformation ability of rock is gradually improved.

In this study, the variation law of different equivalent shear stress *mσ*(*α*) on planes 1–2, 1–3, and 2–3 under TTT conditions are compared (taking *σ*_1_ = 100 MPa, *σ*_3_ = 10 MPa, *σ*_2_ = 20–60 MPa as example). The equivalent shear stress *mσ*(*α*) on plane 1–3 is significantly higher than that of plane 1–2 (Fig. 20b), indicating that the microcracks on plane 1–3 are more likely to expand than those of plane 1–2; therefore, the deformation of rock along the direction of *σ*_3_ is significantly higher than along the *σ*_3_ direction.

The equivalent shear stress *mσ*(*α*) in plane 1–2 decreases gradually but increases in plane 2–3 with increasing *σ*_2_, indicating that the increase of *σ*_2_ gradually inhibits the development of cracks in plane 1–2, while it promotes rock crack growth along the direction of *σ*_2_ (plane 2–3). It can be concluded that when *σ*_2_ is small (20, 30, or 40 MPa), the crack mainly expands on plane 1–3, several cracks expand on the plane1–2, and only a small number of cracks in the direction of *σ*_2_ (plane 2–3) exceed the critical value [*mσ*(*α*)]. Therefore, the increasing *σ*_2_ can inhibit the internal cracks of the rock from expansion in plane 1–2 and plane 2–3, and play a lateral constraint on the rock deformation. When *σ*_2_ is high (50 and 60 MPa), the equivalent shear stress *mσ*(*α*) of the cracks is very small in plane 1–2; consequently, the crack does not substantially expand. The crack propagates in plane 1–3, while a large number of cracks in the direction of *σ*_2_ (in plane 2–3) exceed the critical value [*mσ*(*α*)], and the crack rapidly spreads in the direction of *σ*_2_. As a result, the deformation of the rock is accelerated in the *σ*_2_ direction.

## Conclusion

In this study, a series of conventional triaxial and true triaxial compression tests for sandstone was conducted using a self-developed true triaxial compression test system combined with the AE testing technique. The deformation characteristic and the law of strain in the direction of three principal stresses during the progressive damage of the rock were analyzed. The conclusions can be summarized as follows:

1. Significant differences were observed in the stress–strain curves of the rock under two loading conditions of CTT and TTT conditions, respectively. The axial and lateral strains of rock before failure under CTT conditions increased with increasing confining pressure. With increasing *σ*_2_ under TTT conditions, the axial strain *ε*_1_ and lateral strain *ε*_2_ gradually decreased, and the lateral strain (expansion) of rock is mainly focused along the direction of *σ*_3_. When *σ*_2_ is 20, 30, or 40 MPa, the lateral strain *ε*_3_ and lateral strain difference |*ε*_2_ − *ε*_3_| gradually decreased with increasing, and the anisotropic characteristics gradually weakened. When *σ*_2_ was 50 MPa or 60 MPa, the lateral strain *ε*_3_ and lateral strain difference |*ε*_2_ − *ε*_3_| increased noticeably and the anisotropic characteristics of rock significantly improved. Furthermore, the effect of *σ*_2_ gradually evolved from lateral confinement to promoting rock expansion.
2. In this study, the deformation characteristics of rock in each stage under CTT and TTT conditions were analyzed during the progressive damage process. During the elastic stage II, the confining pressure significantly affected the axial strain, while the effect of confining pressure on the lateral strain remained small. The elastic modulus of rock increased with increasing confining pressure under CTT conditions, increasing first and then decreasing with increasing *σ*_2_ under TTT conditions.
3. The variation of the volume strain increment Δ*ε*_v_ and volume strain rate ∂*ε*_v_/∂*t* of rock during stable and unstable crack growth stages were analyzed in detail. During the stable crack growth stage III, the rock is compressed both under CTT and TTT conditions, while the variation law differed. Under CTT conditions and with increasing confining pressure, Δ*ε*_v_ of rock increases linearly, and the ∂*ε*_v_/∂*t* also increases. However, under TTT conditions with increasing *σ*_2_, both Δ*ε*_v_ and ∂*ε*_v_/∂*t* of the rock specimen decreased after the increase. In contrast, during the unstable crack growth stage IV, the rock expanded under CTT and TTT conditions, while the variation law differed. Under CTT conditions with increasing confining pressure, Δ*ε*_v_ increased sharply, and faster compared to stage III. The volume strain rate ∂*ε*_v_/∂*t* gradually slowed down, and the trend is opposite to that of stage III. Under TTT conditions, when *σ*_2_ is 20, 30, or 40 MPa, Δ*ε*_v_ of rock increased, and ∂*ε*_v_/∂*t* gradually slows down with increasing *σ*_2_. When *σ*_2_ is 50 and 60 MPa, Δ*ε*_v_ of rock gradually decreased, and ∂*ε*_v_/∂*t* clearly accelerated with increasing *σ*_2_.
4. In this study, AE was used to detect the progressive development of micro-cracks in rock, and a 3D rock model with microcrack defects was established. Under CTT conditions with increasing confining pressure, the AE activity showed a “gradually decreasing” trend, the internal cracks of the rock were gradually suppressed, rock deformation slowed down, and the rock’s capacity to bear lateral expansion was strengthened. Under TTT conditions, when *σ*_2_ was 20, 30, or 40 MPa with increasing *σ*_2_, the AE activity weakened, and the internal micro-cracks in the planes parallel to the plane 1–2 and plane 2–3 (planes perpendicular to *σ*_3_ and *σ*_1_) in the rock were gradually constrained, acting as a lateral constraint on rock deformation. For *σ*_2_ of 50 or 60 MPa, with increasing *σ*_2_, the AE activity increased, and the internal micro-cracks development in the planes along the direction of *σ*_2_ accelerated, leading to an acceleration of the lateral deformation of rock. As a result, the rock rapidly formed the macroscopic fracture surface, with a greatly decreased ability to withstand deformation.
